# How accurate is automated gap filling of metabolic models?

**DOI:** 10.1186/s12918-018-0593-7

**Published:** 2018-06-19

**Authors:** Peter D. Karp, Daniel Weaver, Mario Latendresse

**Affiliations:** Bioinformatics Research Group, SRI International, 333 Ravenswood Ave, Menlo Park, 94025 USA

**Keywords:** Flux balance analysis, FBA, Gap-filling, Evaluation

## Abstract

**Background:**

Reaction gap filling is a computational technique for proposing the addition of reactions to genome-scale metabolic models to permit those models to run correctly. Gap filling completes what are otherwise incomplete models that lack fully connected metabolic networks. The models are incomplete because they are derived from annotated genomes in which not all enzymes have been identified. Here we compare the results of applying an automated likelihood-based gap filler within the Pathway Tools software with the results of manually gap filling the same metabolic model. Both gap-filling exercises were applied to the same genome-derived qualitative metabolic reconstruction for *Bifidobacterium longum* subsp. *longum* JCM 1217, and to the same modeling conditions — anaerobic growth under four nutrients producing 53 biomass metabolites.

**Results:**

The solution computed by the gap-filling program GenDev contained 12 reactions, but closer examination showed that solution was not minimal; two of the twelve reactions can be removed to yield a set of ten reactions that enable model growth. The manually curated solution contained 13 reactions, eight of which were shared with the 12-reaction computed solution. Thus, GenDev achieved recall of 61.5% and precision of 66.6%. These results suggest that although computational gap fillers are populating metabolic models with significant numbers of correct reactions, automatically gap-filled metabolic models also contain significant numbers of incorrect reactions.

**Conclusions:**

Our conclusion is that manual curation of gap-filler results is needed to obtain high-accuracy models. Many of the differences between the manual and automatic solutions resulted from using expert biological knowledge to direct the choice of reactions within the curated solution, such as reactions specific to the anaerobic lifestyle of *B. longum*.

**Electronic supplementary material:**

The online version of this article (10.1186/s12918-018-0593-7) contains supplementary material, which is available to authorized users.

## Background

Constructing metabolic models requires the creation of connected network paths from the nutrient compounds that fuel an organisms growth to the end products of the biosynthetic machinery of the organism that comprise the biomass of the organism (the biomass metabolites). However, because the reactions within most metabolic models are derived from the enzyme annotations assigned to genes within a sequenced genome, and because methods for genome annotation are incomplete and fail to assign functions to many genes (and in some cases assign incorrect functions), genome-derived metabolic networks usually contain multiple gaps (missing reactions).

Because filling these gaps manually can take months of effort, computational gap-filling methods have been developed [1–5]. They propose new reactions to add to a metabolic model from an external database of reactions, to enable the production of all biomass metabolites from the supplied nutrient compounds. As the pace of model building quickens, and as researchers strive to build models for microbial communities by integrating many individual models for single organisms [6], model builders have come to rely more and more heavily on these automated gap fillers.

However, there have been few past studies of the accuracy of gap-filling methods. Two studies [2, 6] evaluated the accuracy of their overall reconstruction methodology by evaluating the ability of the final models to predict reaction and gene essentiality, respectively. However, these evaluations did not focus on gap filling alone; they spanned gap filling plus additional methods for supplementing the metabolic network. Reaction and gene essentiality data cannot be used to evaluate the accuracy of gap filling itself because by definition, every reaction added to gap-fill a network is essential to the production of some biomass metabolite (if the reaction were inessential, then adding it to the network would be unnecessary). Two other studies evaluated gap filling itself [3, 4], but those methods supplement basic reaction gap filling with sequence-similarity searches, phylogenetic profiles, and gene-expression data (which is not available for many organisms). Furthermore, the MIRAGE program [3] “aims to resolve all gap-filling problems rather than just enabling biomass production.” — For example, MIRAGE aims to add enough reactions to a model to activate all reactions in the metabolic network (such as those with dead-end metabolites), even if those reactions are not required for biomass production. Hence, its performance results will not be comparable to most gap fillers that aim to enable biomass production (by resolving all gap-filling problems we believe the authors mean enabling production of all dead-end metabolites).

We chose to evaluate the accuracy of basic reaction gap filling without sequence or other information, as a baseline for future studies. Specifically, we address the question of how accurately does a specific gap-filling algorithm predict the correct set of reactions within an organism’s metabolic network? We address this question directly by comparing a manually curated metabolic model for *Bifidobacterium longum* subsp. *longum* JCM 1217 with an automatically gap-filled metabolic model for the same strain. In both cases, we began with the same genome sequence; we annotated that sequence (gene finding and gene-function prediction) using KBase; and we ran the GenBank entry produced by the KBase annotator through Pathway Tools [7, 8] to create a new Pathway/Genome Database (PGDB) containing the predicted reactome and metabolic pathways (the qualitative metabolic reconstruction) of the organism. The resulting PGDB, which we call the *gapped PGDB,* was the input to both a manual gap-filling procedure by an experienced model builder and an automated gap-filling procedure by the GenDev gap filler within the MetaFlux metabolic modeling component of the Pathway Tools software [8].

Our approach is limited by our examination of one metabolic model; we hope other groups will publish similar studies for other models. In addition, we consider only one gap-filling algorithm. Note that comparison of different gap-filling tools will be complicated by the fact that each tool is probably dependent on its own reaction database, and those different reaction databases will vary in size and quality, which will strongly influence gap-filling results.

## Results

The gapped PGDB resulted from the execution of Methods Steps 1–5 and contained 1121 reactions catalyzed by 523 enzymes, 104 metabolic pathways, 914 metabolites, and 91 transport reactions. This gapped reaction network was capable of producing 15 of the 53 biomass metabolites defined for the model (see Table 1), from four nutrient compounds (MetaFlux can identify what subset of biomass metabolites can be produced by a given set of reactions and nutrients).

**Table 1:** Biomass metabolites defined for the *B. longum* model. An “X” in column 3 identifies metabolites that could be produced by the model before gap filling was performed

Name	BioCyc ID	Pre gap fill?
(2E,6E)-farnesyl diphosphate	FARNESYL-PP	
1-deoxy-D-xylulose 5-phosphate	DEOXYXYLULOSE-5P	
2-C-methyl-D-erythritol 4-phosphate	2-C-METHYL-D-ERYTHRITOL-4-PHOSPHATE	
2-C-methyl-D-erythritol-2,4-cyclodiphosphate	2C-METH-D-ERYTHRITOL-CYCLODIPHOSPHATE	
2-phospho-4-(cytidine 5’-diphospho)-2-C-methyl-D-erythritol	2-PHOSPHO-4-CYTIDINE-5-DIPHOSPHO-2-C-MET	
ADP	ADP	X
ADP-alpha-D-glucose	ADP-D-GLUCOSE	X
AMP	AMP	X
ATP	ATP	X
CDP-1,2-dipalmitoylglycerol	CPD-12815	
CTP	CTP	
GMP	GMP	X
GTP	GTP	X
H+	PROTON	X
H2O	WATER	X
L-alanine	L-ALPHA-ALANINE	
L-arginine	ARG	
L-asparagine	ASN	
L-aspartate	L-ASPARTATE	
L-cysteine	CYS	
L-glutamate	GLT	
L-glutamine	GLN	
L-histidine	HIS	
L-isoleucine	ILE	
L-leucine	LEU	
L-lysine	LYS	
L-methionine	MET	
L-phenylalanine	PHE	
L-proline	PRO	
L-serine	SER	
L-threonine	THR	
L-tryptophan	TRP	
L-tyrosine	TYR	
L-valine	VAL	
NAD+	NAD	X
NADH	NADH	X
NADP+	NADP	X
NADPH	NADPH	X
S-adenosyl-L-methionine	S-ADENOSYLMETHIONINE	X
UDP-N-acetyl-alpha-D-glucosamine	UDP-N-ACETYL-D-GLUCOSAMINE	
C1	C1	
UTP	UTP	
chorismate	CHORISMATE	X
dATP	DATP	
dCTP	DCTP	
dGTP	DGTP	
dTTP	TTP	
di-trans,octa-cis-undecaprenyl diphosphate	UNDECAPRENYL-DIPHOSPHATE	
dipalmitoyl phosphatidate	CPD0-1422	
C6	C6	
glycine	GLY	
isopentenyl diphosphate	DELTA3-ISOPENTENYL-PP	
phosphate	Pi	X

C1 = UDP-N-acetyl-alpha-D-muramoyl-L-alanyl-gamma-D-glutamyl-meso-2,6-diaminopimeloyl-D-alanyl-D-alanine. C6 = ditrans,octacis-undecaprenyldiphospho-N-acetyl-(N-acetylglucosaminyl)muramoyl-L-alanyl-gamma-D-glutamyl-meso-2,6-diaminopimeloyl-D-alanyl-D-alanine

The MetaFlux gap filler, GenDev (see “Methods” section), computed a minimum-cost solution to the problem of gap-filling the network: it proposed adding 12 new reactions to the PGDB (see Table 2), which resulted in production of all 54 of the biomass metabolites via 241 reactions (including the new reactions) carrying non-zero flux. GenDev is a parsimony-based gap filler that seeks minimum-cost solutions. For this solution all reactions added by GenDev had the same cost because the taxonomic range and directionality information stored in MetaCyc for all added reactions were compatible with *B. longum*.

**Table 2:** Reactions added to the model by the automated gap filler GenDev (marked “A” in the last column) and by manual gap filling (marked “M” in the last column). Reactions marked “*” in the last column are excess reactions predicted by GenDev that are not in fact needed for growth of the model

BioCyc Reaction ID	Reaction	Added By
ASNSYNA-RXN	L-aspartate + ammonium + ATP →L-asparagine + AMP + PPi + H+	A
RXN-1381	palmitoyl-CoA + sn-glycerol 3-phosphate →	A
	1-palmitoylglycerol 3-phosphate + coenzyme A	
DUDPKIN-RXN	dUDP + ATP →dUTP + ADP	A,*
CDPKIN-RXN	CDP + ATP →CTP + ADP	A,*
DTDPKIN-RXN	dTDP + ATP →dTTP + ADP	A,M
UDPKIN-RXN	UDP + ATP →UTP + ADP	A,M
GPPSYN-RXN	dimethylallyl diphosphate + isopentenyl diphosphate →	A,M
	geranyl diphosphate + diphosphate	
FPPSYN-RXN	geranyl diphosphate + isopentenyl diphosphate →	A,M
	(2E,6E)-farnesyl diphosphate + diphosphate	
RXN-8999	(2E,6E)-farnesyl diphosphate + 8 isopentenyl diphosphate →	A,M
	di-trans,octa-cis-undecaprenyl diphosphate + 8 diphosphate	
IGPSYN-RXN	1-(o-carboxyphenylamino)-1’-deoxyribulose 5’-phosphate + H+ →	A,M
	(1S,2R)-1-C-(indole-3-yl)glycerol 3-phosphate + CO2 + H2O	
HISTIDPHOS-RXN	L-histidinol-phosphate + H2O →histidinol + phosphate	A,M
PREPHENATE-TRANSAMINE-RXN	L-arogenate + 2-oxoglutarate →prephenate + L-glutamate	A,M
RXN-12460	an L-asparaginyl-[tRNAasn] + H2O →L-asparagine + tRNAasn + H+	M
GDPKIN-RXN	GDP + ATP →GTP + ADP	M
GLUCOKIN-RXN	D-glucopyranose + ATP →D-glucopyranose 6-phosphate + ADP + H+	M
RXN-17018	a palmitoyl-[acp] + sn-glycerol 3-phosphate →	M
	1-palmitoylglycerol 3-phosphate + a holo-[acyl-carrier protein]	
RXN-17897	2 an oxidized ferredoxin [iron-sulfur] cluster + NADPH →	M
	2 a reduced ferredoxin [iron-sulfur] cluster + NADP+ + H+	

However, we determined that 2 of the 12 reactions added by GenDev (marked by “*” in Table 2) were not in fact required for production of the 54 biomass metabolites. We determined this by iteratively removing reactions from the GenDev result set of 12 reactions and then checking using flux balance analysis (FBA) whether the model would grow. The non-minimum gap-filling solution returned by GenDev is due to numerical imprecision in the mixed-integer linear programming (MILP) solver, SCIP (http://scip.zib.de/); we explore these issues in more detail in [9]. Similar numerical imprecision issues are discussed in [10, 11]. The ten reactions marked with “A” but not “*” do constitute a minimum set of reactions for enabling model growth.

Thirteen metabolic reactions were added to the model during manual gap filling. Eight reactions (marked as “A,M” in Table 2) are common between the 12 reactions added by GenDev and the 13 flux-carrying reactions added by manual gap filling.

We evaluate the accuracy of GenDev as follows:

- true positives (tp) = 8
- false positives (fp) = 4
- false negatives (fn) = 5
- Recall = 61.5% = tp / (tp + fn)
- Precision = 66.6% = tp / (tp + fp)

Let us consider the four reactions predicted by GenDev but not by manual gap filling:

- CDPKIN-RXN: This reaction is a complicated case. It is in fact not needed to enable growth of the model; therefore we treat it as a false-positive prediction, probably due to a numerical error in the MILP solver. However, it was present in our manually curated model because its presence gives correct flux rates; see Discussion for more details.
- DUDPKIN-RXN: This reaction is in fact not needed to enable growth of the model; it is a false-positive prediction, probably due to a numerical error in the MILP solver.
- RXN-1381 (GenDev) and RXN-17018 (manual) are very similar to one another — the former cleaves palmitoyl-CoA, whereas the latter cleaves the class “a palmitoyl-[acp]” — since they do not match exactly we count this result as a false-positive prediction.
- ASNSYNA-RXN: This reaction is one of *four* reactions in MetaCyc that, if added to the model, enables production of L-asparagine. The correct reaction to add is RXN-12460, because of the presence in *B. longum* of the two other enzymes in the MetaCyc pathway L-asparagine biosynthesis III (tRNA-dependent) (PWY490-4). These four reactions have equal cost, thus the gap filler is randomly selecting among them.

The following five reactions were predicted to be present by manual gap filling but not by GenDev:

- GDPKIN-RXN: In the manually curated model, a GDP kinase activity, GDPKIN-RXN, forms GTP from GDP. In the GenDev model, pyruvate kinase RXN-14117 is instead used to transfer phosphate from phosphoenolpyruvate to GDP. There is no clear dedicated NDP kinase in the *B. longum* annotation and no hits to Pfam PF00334 in the genome, but there is an adenylate kinase at blongannot.CDS.3717, 1 and adenylate kinases have been observed to have substantial NDP kinase activity capable of complementing *ndk* knockout [12, 13]. Pyruvate kinase has also been demonstrated to transfer phosphate from phosphoenolpyruvate to GDP [14, 15]. Both mechanisms are plausible means of phosphorylating GDP, but because NDP kinase activity is likely required to regulate the nucleotide pool balance, we believe that GDPKIN-RXN is the preferable choice.
- GLUCOKIN-RXN: This reaction was originally included as a model-building convenience and used to convert glucose (GLC) supplied in the cytosol to glucose 6-phosphate for entry into the glycolysis reactions. We then identified a polyphosphate-based glucokinase activity (EC 2.7.1.63) in the KBase annotation at gene blongannot.CDS.3457. MetaCyc contains a polyphosphate glucokinase reaction (2.7.1.63-RXN) but GenDev cannot include polymerization/depolymerization reactions such as
  2.7.1.63-RXN during gap filling, so GLUCOKIN-RXN remains as a way of marshaling polyphosphate reserves. Glucose can enter the cell through the glucose/galactose transporter at blongannot.CDS.3321 (PF07690.14 Major Facilitator Superfamily bitscore 52.89) and the phosphotransferase system (PTS) elements present at blongannot.CDS.2260 and blongannot.CDS.2261. The automated GenDev solution uses the PTS-based transport reactions for glucose and does not show glucokinase activity.
- RXN-17018 is very similar to RXN-1381 inferred by GenDev.
- RXN-12460: See preceding discussion of ASNSYNA-RXN.
- RXN-17897: We added this reaction to MetaCyc and the *B. longum* PGDB to represent cytoplasmic ferredoxin-NADP+ reductase activity (EC 1.18.1.2) carried out by the blongannot.CDS.2132 product, since the existing MetaCyc reaction is a membrane reaction. PSORTdb [16] classifies this protein as cytoplasmic with 99.5% likelihood. This reaction enables transfer of reducing equivalents from NADPH to ferredoxin to provide ferredoxin for the MEP pathway and recycling of the ferredoxin pool. GenDev reverses RXN0-882 to run a futile cycle that regenerates the reduced ferredoxin required in ISPH2-RXN (see Table 3), but RXN0-882 (see Table 3) is not physiologically reversible. RXN0-882 is an example of a MetaCyc reaction with an unset direction whose physiological direction is not correctly inferred in a PGDB context.

**Table 3:** Additional discussed reactions

BioCyc Reaction ID	Reaction
ISPH2-RXN	isopentenyl diphosphate + 2 oxidized ferredoxin [iron-sulfur] cluster + H2O →
	(E)-4-hydroxy-3-methylbut-2-en-1-yl diphosphate + 2 reduced ferredoxin [iron-sulfur]
	cluster + H+
RXN0-882	(E)-4-hydroxy-3-methylbut-2-en-1-yl diphosphate + 2 oxidized ferredoxin [iron-sulfur]
	cluster + H2O →
	2-C-methyl-D-erythritol-2,4-cyclodiphosphate + 2 reduced ferredoxin [iron-sulfur]
	cluster + H+
RXN0-302	2-phospho-4-(cytidine 5’-diphospho)-2-C-methyl-D-erythritol →2-C-methyl-D-erythritol-
	2,4-cyclodiphosphate + CMP

## Discussion

We describe a comparison of automated and manual metabolic-model gap-filling methods applied to a gapped PGDB for *B. longum*. The methods started with the same initial reaction network: a qualitative metabolic reconstruction computed by the PathoLogic component of Pathway Tools that underwent some subsequent curation — mostly the addition of reactions based on manual review of enzyme names that were not recognized by PathoLogic.

Three important restrictions on automatic gap-filling methods should be mentioned. Gap-filling methods drawing from a reaction database (MetaCyc, in our case) can only operate within the space of knowledge contained in that database. If the reaction database does not contain a needed reaction, as for RXN-17897 in our work, then an automatic gap filler could not include that reaction in its solution set. Similarly, if appropriate compound instances are not present in the relevant PGDB, then the automatic gap filler could not include instantiated reactions containing those compounds in its solution set. These restrictions, along with the need to verify genome-annotation quality, suggest continued importance for human curation of reaction databases and supervision of automatic model-building processes.

We found that GenDev did not return solutions that were minimum cost; in our *B. longum* example, its 12-reaction solution contained two extra, unneeded reactions. Based on more detailed studies of this topic [9], we believe these extra reactions were present because of numerical imprecision in the solver. Such extra reactions occur in approximately 10% of gap-filler runs with solutions on the order of 10 reactions using the SCIP solver.

This result suggests cause for concern that larger solutions containing more reactions (e.g., Henry et al. [2] added an average of approximately 60 gap-filled reactions to their models) may contain even more unneeded reactions. Numerical imprecision arises from linear constraints that control the addition of candidate reactions to the model or from the linear constraints to maintain the steady state. The former constraints have the form

1 $$ B_{l} s_{r} \leq f_{r} \leq B_{u} s_{r}   $$

where the Boolean variable *s*_*r*_ has value 0 or 1 to control the addition of candidate reaction *r*; variable *f*_*r*_ is for the flux of candidate reaction *r*; and constants *B*_*l*_ and *B*_*u*_ are the lower and upper bounds, respectively, for the flux of any reaction considered to be active. In some cases, *s*_*r*_ is set to 0 (that is, saying not to add these reactions); but in fact, their fluxes *f*_*r*_ were all equal or above *B*_*l*_, violating the constraints. In such a case, despite what the variables *s*_*r*_ say, the candidate reactions are suggested to be added, because in general, such reactions are likely needed to obtain growth. Such cases can be detected and are reported by MetaFlux, which did not occur in this work. On the other hand, the constraints maintaining the steady state can have cumulative numerical imprecision that is much harder to detect, and where a reaction is erroneously made active to satisfy them and the constraint that the biomass flux must be greater than 10^−3^. Such cases are not detected by MetaFlux.

Our results also call into question the basic assumption that gap fillers should seek solutions containing a minimal number of reactions. Our manually curated solution contains 13 reactions compared to the minimum solution of 10 reactions — a 30% increase over the minimum solution. This result supports our design of GenDev as computing a *minimal-cost* solution, where cost reflects not simply the number of reactions added, but also factors such as changes to the direction of existing reactions, and whether the organism in which a reaction is inserted is within the expected taxonomic range of the reaction.

Further analysis revealed that although we had thought this manual solution to be minimal, it was not. If we remove CDPKIN-RXN from the model, the model still grows. This solution is minimal, but the model grows 45% more slowly without this reaction. The model secretes CMP because it can no longer recycle CMP produced from RXN0-302 (see Table 3) in pathway NONMEVIPP-PWY (methylerythritol pathway I). This example illustrates the point that although gap fillers may enable growth of a model, they do not ensure that the model grows at the correct rate, or that the model sends the correct relative flux rates through different pathways. Manual model curation is currently the only method that can satisfy these criteria.

Observed differences between the manual solution and the automatic gap-filled solution arose from our ability to evaluate preferences among different potential reactions to add based on expert biological knowledge. For example:

- We used GDP kinase activities to form GTP, as opposed to a pyruvate kinase activity relied upon by the gap-filled model, because of our strong expectation that NDP kinase activity exists to regulate nucleotide pool balances.
- We used a mixed glucokinase and PTS-based approach to introduce glucose 6-phosphate into the cell, based on observation of polyphosphate-based glucokinase in the genome annotation, whereas the gap-filled solution used only a PTS-based approach.
- The manually gap-filled solution uses class III ribonucleotide reductase reactions [17] to form dNTPs from their nucleotide equivalents, whereas the automatic gap-filling approach uses thioredoxin to form dNDPs from NDPs in a class Ia RNR reaction. *B. longum* contains *nrdHIEF* (glutaredoxin-like RNR class Ib) and *nrdDG* (RNR class III) gene clusters. Based on *B. longum*’s anaerobic lifestyle the strictly anaerobic class III RNR is likely to play a prominent role and was chosen for the manual solution.
- We added the new reaction RXN-17897 to ensure availability of reduced ferredoxin for the isoprenoid pathway.
- We added reaction RXN-12460 — marked as a hypothetical reaction in MetaCyc — because of the presence of other enzymes in its pathway. Note that had we used the default pathway prediction behavior in PathoLogic, this reaction and pathway would have been imported by PathoLogic. However, we used a stringent setting in which a pathway had to have enzymes for all of its reactions present for the pathway to be inferred. Since no enzyme is known for hypothetical reactionRXN-12460, the pathway was not inferred. In the future we will modify PathoLogic to not require the presence of enzymes for hypothetical reactions.

In some cases, multiple MetaCyc reaction objects describe the same chemical transformation. This is most commonly the case with chemical transformations that are described by both an object denoting a specific reaction and an object denoting a generic reaction that can be instantiated to that specific reaction. For example, one reaction may specifically describe hydrolysis of ATP to ADP and phosphate, while another reaction hydrolyzes NTPs to NDPs and phosphate. This situation can lead to semantically redundant alternative solutions that complicate the user’s task of reviewing and understanding the alternative solutions that may be generated across multiple executions of the gap filler.

Lack of an appropriate cytosolic-only electron transfer from NADPH to ferredoxin in MetaCyc caused us to create the new reaction RXN-17897 to satisfy a requirement for reduced ferredoxin in the isoprenoid pathway. The existing MetaCyc activity involved membrane transport as part of the reaction. Manual reaction network curation plays an important role in repairing problems of this kind, since automatic gap-filling methods cannot yet suggest new reaction activities to address network problems.

We urge other groups to perform similar analyses to that presented herein for other genomes and for other gap-filling software. Comparison of different gap-filling programs will be problematic unless all programs use the same reference reaction database (e.g., MetaCyc). However, modifying various programs to use the same reference database may be quite time consuming.

## Methods

We compared manual gap filling with automated gap filling by applying both gap-filling approaches to the same starting PGDB. The following steps yielded that starting PGDB from the nucleotide sequence of *B. longum*.

1. The nucleic acid sequence of *B. longum* was obtained from GenBank record NC_015067.1.
2. The *B. longum* sequence was submitted to the KBase microbial genome annotation pipeline for gene finding and protein-function prediction. 2172 genes were identified (2043 protein-coding genes). The resulting annotation was exported from KBase to a GenBank-format file. We did not use the original annotation present in GenBank record NC_015067.1 because the KBase annotation contained significantly more enzyme functions; which annotation is more accurate, we do not know.
3. That GenBank-format file (see Additional file 1) was processed by the PathoLogic module [18] of Pathway Tools (a pre-release version of version 22.0) to create an initial *B. longum* PGDB, BlongCyc. A very strict pathway score cutoff of 1.0 was supplied to PathoLogic to predict into BlongCyc (from MetaCyc) only the pathways that have gene annotations associated with all pathway reactions, to minimize the effects of pathway inference on biomass goal reachability. PathoLogic inference of a metabolic pathway causes all reactions within the pathway to be imported from the MetaCyc database into the new PGDB, including reactions lacking gene assignments — using the 1.0 cutoff means that no reactions lacking gene assignments were imported from MetaCyc during pathway inference. The resulting PGDB was subjected to the following manual refinement steps. That is, some manual refinement occurred before gap filling began.
4. Probable enzyme assignment was performed, whereby genes in the annotated genome that had not been assigned to reactions by PathoLogic were examined manually and in some cases assigned to reactions that were manually imported from MetaCyc. Such manual gene–reaction assignments were performed if (a) there existed a human-recognizable name match and a match of the first three sections of the EC number between the assignment and MetaCyc, or (b) the best HMMER3 TIGRFAM hit to the sequence of the probable enzyme had a bitscore of higher than 100 and a GO molecular functional annotation corresponding to a MetaCyc reaction.
5. BlongCyc reactions were curated to explicitly specify reversibility to avoid inconsistencies arising from automatic reaction direction inference. Twenty-eight reactions (see Table 4) were set to be either unidirectional or reversible according to literature research and the energetic considerations; these directionality changes were also set in MetaCyc to improve directionality inference by PathoLogic in the future.

**Table 4:** *B. longum* and MetaCyc reactions whose directionality was manually curated during *B. longum* model development. Reaction identifiers can be mapped to actual reactions at the MetaCyc.org website

BioCyc Reaction ID	Metabolic Goal	Curated Direction
METHENYLTHFCYCLOHYDRO-RXN	Glycine and one-carbon metabolism	REVERSIBLE
HYPOXANPRIBOSYLTRAN-RXN	Histidine synthesis	PHYSIOL-R2L (PPi produced)
IMP-DEHYDROG-RXN	Xanthine synthesis	REVERSIBLE
RIBOKIN-RXN	Ribose synthesis	PHYSIOL-L2R (kinase activity)
RXN-14223	Ribose degradation	PHYSIOL-L2R
TYROSINE-AMINOTRANSFERASE-RXN	Tyrosine synthesis	REVERSIBLE (aminotransferase activity)
GLYC3PDEHYDROGBIOSYN-RXN	Phospholipid synthesis	PHYSIOL-R2L (avoid spontaneous
		NADH → NADPH transhydrogenation)
1.1.1.8-RXN	Phospholipid synthesis	PHYSIOL-R2L (avoid spontaneous
		NADH → NADPH transhydrogenation)
MALONYL-COA-ACP-TRANSACYL-RXN	Phospholipid synthesis	PHYSIOL-L2R (biosynthetic direction)
3-OXOACYL-ACP-REDUCT-RXN	Phospholipid synthesis	PHYSIOL-R2L (avoid NADPH-generating cycle)
RXN0-6705	Phospholipid synthesis	PHYSIOL-R2L (avoid NADPH-generating cycle)
PHOSPHAGLYPSYN-RXN	Phospholipid synthesis	PHYSIOL-L2R (avoid NADPH-generating cycle)
H2PTERIDINEPYROPHOSPHOKIN-RXN	THF synthesis	PHYSIOL-L2R
RXN-9772	NAD synthesis	PHYSIOL-L2R
RIBULP3EPIM-RXN	Pentose phosphate pathway	REVERSIBLE
RIB5PISOM-RXN	Pentose phosphate pathway	REVERSIBLE
1TRANSKETO-RXN	Pentose phosphate pathway	REVERSIBLE
2TRANSKETO-RXN	Pentose phosphate pathway	REVERSIBLE
TRANSALDOL-RXN	Pentose phosphate pathway	REVERSIBLE
PHOSACETYLTRANS-RXN	Central carbon metabolism	REVERSIBLE
RXN-12195	Phosphorylation debugging	PHYSIOL-L2R
RXN-12196	Phosphorylation debugging	PHYSIOL-L2R
XANPRIBOSYLTRAN-RXN	Phosphorylation debugging	PHYSIOL-R2L
DCTP-PYROPHOSPHATASE-RXN	Phosphorylation debugging	PHYSIOL-L2R
RXN-7913	Phosphorylation debugging	PHYSIOL-L2R
ATPASE-RXN	Phosphorylation debugging	PHYSIOL-L2R
RXN0-5468	Phosphorylation debugging	PHYSIOL-L2R
ALDHDEHYDROG-RXN	Phosphorylation debugging	PHYSIOL-L2R
6. The Pathway Tools Transport Inference Parser [19] was used to infer transport reactions from transporter names, followed by additional manual assignments of same.

Correct reaction directionality is essential for proper operation of a metabolic model, because of the paramount importance of energy-consuming reactions in determining the overall direction of flux in the metabolic reaction network. In MetaFlux, PGDB reactions with unspecified direction have their direction inferred by MetaFlux during execution of the model based on the direction(s) in which the reaction occurs in one or more pathways. We found that this inference process added a significant degree of unpredictability to reaction directions. We therefore explicitly specified reaction directions in both *B. longum* and the MetaCyc database whenever ambiguous reactions were encountered during manual model curation.

Consequently, we specified reaction directionality for all reactions involved in the model and propagated these specifications to MetaCyc to improve the quality of subsequent models constructed in PathoLogic. In deciding on reaction directionality and reversibility, we follow the general advice given by Thiele and Palsson [20]: ATP formation should be associated only with recognized substrate-level phosphorylation reactions and the activity of ATP synthase, and NADH should not reduce NADP+ without an energetic coupling. We find that these two simple rules are appropriate for deciding a very large majority of reaction-direction questions. They ensure biochemically relevant fluxes through oxidative pathways. The central role of ferredoxin-mediated NADP+ reduction in *B. longum* biosynthetic metabolism made it especially important to carefully curate routes to NADPH in this work.

A minimal set of nutrients, biomass metabolites, and waste products for *B. longum* was identified through literature research. This research yielded a simple nutrient set (glucose, ammonium, inorganic phosphate, and hydrogen sulfide); 53 core biomass metabolites (amino acids, phosphorylated nucleotides, isoprenoids, phospholipids, sugar nucleotides, NAD(P), SAdoMet, UDPNacGlc, and undecaprenyl diphosphate) and 8 fermentative waste products (protons, water, carbon dioxide, fumarate, succinate, acetate, formate, and lactate). These metabolites defined the inputs and outputs of the *B. longum* metabolic model.

### Automated gap filling

We used the MetaFlux module of Pathway Tools (a pre-release version of version 22.0) to perform automated gap filling of the BlongCyc PGDB produced from step (5) above. We used the Technique C variant of the full MetaFlux gap filler [7, 21] (henceforth, *GenDev* — also known as General Development Mode). Technique C is described in detail in [9]. GenDev uses Mixed-Integer Linear Programming (MILP) to find a minimum-cost set of reactions to add to the *B. longum* PGDB from MetaCyc [22] that enable production of all biomass metabolites specified for the PGDB. The cost function defines a set of weights for modifying the existing reactions in the PGDB and for inserting new reactions into the PGDB from MetaCyc. For example, the cost of reversing a reaction already in the *B. longum* PGDB is less than the cost of inserting a new reaction from MetaCyc where *B. longum* is *within* the taxonomic range of that reaction, which in turn is less than the cost of inserting a new reaction from MetaCyc where *B. longum* is *outside* the taxonomic range of that reaction.

The gapped PGDB was gap-filled by MetaFlux to enable growth via a biomass objective. In the manual case, the model was completed by manual inference and analysis of the genome, but to enable flux through the same biomass objective. The biomass synthesis objective used in this study was a general and simplified description of bacterial metabolism at the molecular level and can stand in for a similar general self-reproductive biomass demand for many other bacteria besides *B. longum*. To obtain a complete model of *B. longum* metabolism further model refinement is required, including addition of appropriate cell wall polysaccharides and peptidoglycan, phospholipids and other membrane constituents, and assimilatory pathways for common gut polysaccharides.

### Manual Gap Filling

We manually constructed a draft MetaFlux metabolic model for *B. longum* as a reference standard for our automated gap-filling work. This model was constructed with the goal of satisfying the basic metabolic requirements of *B. longum* biomass by fermentation of glucose. These basic requirements are very general to bacteria and can be divided into the following categories: amino acids, RNA, DNA, phospholipids, glycogen, peptidoglycan, essential cofactors, and growth/non-growth-associated ATP requirements.

It took about three weeks to manually gap-fill the reaction network to produce a metabolic model capable of execution by MetaFlux. We employed three main techniques during manual gap-filling to infer the presence of reactions in the metabolic model:

- Manual inference from biochemical requirement, (e.g., a need to synthesize an amino acid)
- Manual inference from text or EC number in the genome annotation
- Hidden Markov Model (HMM) searches against the genome sequence using TIGRFAMS [23]

Our manual model gap-filling process was based on the fundamental principle that the genome must encode a complete capacity for self-reproduction within a basic nutritional context and that:

- The model should successfully reach a basic biomass synthesis objective representing simple metabolic activity at the level of amino acids, nucleotides, lipids, and so on
- It should do so while carrying out a recognizable fermentative anaerobic metabolism and maintaining a membrane potential
- It should maintain biochemically sensible reaction direction in regard to redox and phosphorylation processes, avoiding NADP+ reduction by NADH and ATP formation without coupling to an energy-yielding reaction

Briefly, the metabolic model was constructed by starting from glucose and working forward through the pathways and reactions to reach the goal metabolites, using the reactions catalyzed by identified genes as a guide. We performed curation of the PGDB to repair model gaps by adding compounds required for reaction instantiation that had been skipped in the automatic metabolite-import process, and in some cases, we curated reactions in both the PGDB and MetaCyc to explicitly specify reaction directionality. In a handful of cases, the reactions deemed necessary for metabolic function could not be associated with genes in the *B. longum* genome.

We determined the fermentative and transport behavior of the model based on the reported systematic microbiology of *B. longum* [24]. Transmembrane transport of weak acids was modeled by co-transport of the weak acid anion with sufficient protons to neutralize overall charge. No respiratory pathways were found in the genome, and a strictly fermentative metabolism is modeled. Pathways for biosynthesis of thiamine, nicotinate, and folate are present in this organism.

The one-carbon folate pathway required transfer of several folate compound instances for proper instantiation, because folate compound instances must all be in the same polyglutamation state to pass through the instantiation process correctly. We have previously observed that PathoLogic can fail to import all folate instances required for the folate pathway instantiation, and we encourage MetaFlux model builders working with instantiated reactions to check carefully that all needed compound instances are present for each instantiated reaction.

A large number of manually created model reactions were transport reactions involved with fermentation. Indeed, the single cytosolic metabolic reaction we created, RXN-17897, was created to fix a compartmentalization issue in fermentative electron transfer. The protons carried across the membrane by weak acids play a vital role in creating the transmembrane proton gradient, and proton balancing of transport reactions is essential to producing the correct stoichiometric ratios of fermentative products. We have taken a simple approach of co-transporting enough protons to neutralize the charges on weak acids (one proton for monocarboxylates and two protons for dicarboxylates) except for formate, which we allow to pass through the membrane alone based on FocA channel activity in *E. coli* [25]. Reactions involving active transport of substrates through the membrane, such as glucose, require either ATP hydrolysis or co-transport of a proton down the transmembrane gradient. Additional file 2 defines the nutrients, secreted compounds, and biomass metabolites for the manually curated model. Additional file 3 contains the results of executing the manually curated model.

## Conclusions

In this empirical investigation of gap filling of metabolic networks, we compared a manually curated gap-filling solution with a solution obtained by the GenDev software. The solution computed by GenDev contained 12 reactions, but closer examination showed that solution was not minimal; a ten-reaction subset would enable model growth. The failure to produce a minimal solution was due to numerical imprecision. The manually curated solution contained 13 reactions. The two solutions contained eight reactions in common. Based on these results, GenDev achieved recall of 61.5% (the fraction of reactions predicted from the correct solution) and precision of 66.6% (the fraction of predicted reactions that were correct).

If the results from this study generalize to other applications of automatic gap fillers, they suggest that although computational gap fillers are populating metabolic models with significant numbers of correct reactions, automatically gap-filled metabolic models also contain significant numbers of incorrect reactions. Our conclusion is that manual curation of gap-filler results is needed to obtain high-accuracy models. Although that statement has been made in other publications, it has not been supported by empirical evidence.

We also observed that the manually curated solution contains 30% more reactions (13 reactions) than the minimal automatically gap-filled solution (10 reactions) computed by GenDev, calling into question the minimality objective of gap fillers.

Some of the differences between the manual and automatic solutions resulted from using expert biological knowledge to direct the choice of reactions within the curated solution, such as reactions specific to the anaerobic lifestyle of *B. longum*, and reactions that yielded flux rates for specific reactions within the model that were closer to flux rates measured experimentally. Future work could attempt to capture such expert knowledge within a gap filler, which would require specifying information about the organism’s environment and physiology to the gap filler, and modifying the gap-filler objective function to use this information.

## Additional files

Additional file 1 Curated *B. longum* PGDB suitable for loading into Pathway Tools version 22.0 for browsing and exploration. This archived directory tree will expand to a set of files readable by Pathway Tools version 22.0. (ZIP 2472 kb)

Additional file 2 FBA definition file for curated *B. longum* metabolic model suitable for execution by Pathway Tools version 22.0. This file defines the nutrients, secretions, and biomass metabolites for execution of the model in conjunction with the *B. longum* PGDB. (TXT 69 kb)

Additional file 3 Results of executing the curated *B. longum* metabolic model with Pathway Tools version 22.0. (TXT 67 kb)

## Abbreviations

FastDev: Fast development mode
FBA: Flux balance analysis
GenDev: General development mode
LP: Linear programming
MILP: Mixed-integer linear programming

## Footnotes

1 KBase assigns gene identifiers of the form “blongannot.CDS.nnnn,” which we preserve in the PGDB.
